# Anesthesia Practitioners’ Goals for Sevoflurane Minimum Alveolar Concentration at the End of Surgery and the Incidence of Prolonged Extubations: A Prospective and Observational Study

**DOI:** 10.7759/cureus.63371

**Published:** 2024-06-28

**Authors:** Kaitlyn R Clevenger, Franklin Dexter, Richard H Epstein, Rakesh Sondekoppam, Anil A Marian

**Affiliations:** 1 Anesthesia, University of Iowa, Iowa City, USA; 2 Anesthesiology, University of Miami Miller School of Medicine, Miami, USA

**Keywords:** operations research, economics of healthcare, delayed awakening from anesthesia, inhalational anesthesia, minimum alveolar concentration(mac)

## Abstract

Background: Prolonged times to tracheal extubation (≥15 minutes from dressing on the patient) are consequential based on their clinical and economic effect. We evaluated the variability among anesthesia practitioners in their goals for the age-adjusted end-tidal minimum alveolar concentration of sevoflurane (MAC) at surgery end and achievement of their goals.

Methods: We prospectively studied a cohort of 56 adult patients undergoing general anesthesia with sevoflurane as the sole anesthetic agent, scheduled operating room time of at least 3 hours, and non-prone positioning. At the start of surgical closure, an observer asked the anesthesia practitioner their goal for MAC when the surgical drapes are lowered (i.e., the functional end of surgery for the studied procedures). When the drapes were lowered, the MAC achieved was recorded, and the values were compared.

Results: The standard deviation of the practitioners’ MAC goal was large, 0.199 (N = 56 cases, 95% confidence interval 0.17-0.24), not significantly different from the standard deviation of the MAC achieved of 0.253, P = 0.071. The MAC goal and MAC achieved were correlated pairwise, Pearson r =0.65, P < 0.0001. There was no incremental effect of operating room conversation(s) related to case progress on the association (partial correlation ‑0.01, P = 0.96). Differences among practitioners in the MAC achieved at surgery end were consequential. Specifically, for the N = 12 cases with prolonged extubation, the mean MAC was 0.60 (standard deviation 0.10) versus 0.48 (0.21) among the N = 44 cases without prolonged extubation (P = 0.0070).

Conclusions: The standard deviation of the MAC goal among practitioners was sufficiently large to contribute significantly to the variability in the MAC achieved at the end of surgery. We confirmed prospectively that the age-adjusted end-tidal MAC at the end of surgery matters clinically and economically because differences of 0.60 versus 0.48 were associated with more prolonged extubations. Our novel finding is that the MAC achieved ≥0.60 were caused in part by the anesthesia practitioners’ stated MAC goals when surgical closures started.

## Introduction

Prolonged times to tracheal extubation (≥15 minutes from dressing first applied) [1,2] are consequential based on their association with the risk of reintubation [3], respiratory treatments in the post-anesthesia care unit [3], administration of flumazenil and naloxone [3], poor-quality ratings [4], longer times in the operating room [5], longer workdays [6], and non-anesthesia practitioners in the operating room waiting idly for extubation [7,8]. When the end-tidal volatile agent concentration of a volatile agent (e.g., sevoflurane) is not successfully titrated down from surgical levels toward the concentration required for awakening as the end of surgery approaches, prolonged extubation is more likely to occur. Therefore, the altitude- and age-adjusted end-tidal minimum alveolar concentration (MAC) at the end of surgery is important because of its association with prolonged extubations. Predictions for the MAC, when individual patients will awaken, are inaccurate because (a) the dose response to inhalational agents does not match between induction and maintenance versus emergence because of pharmacodynamic hysteresis and (b) emergence is often an abrupt temporal transition [9-11]. Our primary objective was to quantify variability among anesthesia practitioners for their MAC goal at the end of surgery. In this prospective study, we asked anesthesia practitioners for their MAC goal when the surgical drapes are lowered. We hypothesized that the standard deviation among practitioners in their goals for the MAC would be small when assessed on an absolute basis and smaller, when assessed on a relative basis, compared with the standard deviation among practitioners of the MAC achieved when the surgical drapes were lowered.

Achieving a MAC goal can be challenging for several reasons [8,12]. Surgical closure times have large proportional variability, even greater than the surgical times themselves [8,12]. Times remaining in surgical cases do not decrease like simple countdown timers [8-14]. That is, if there is a prediction, based on historical data, that the case will end in 30 minutes, 15 minutes later, if the case is still ongoing, the predicted time to the end of the case is not 15 minutes but longer [12-14]. Furthermore, with fresh gas flows of approximately one liter per minute, when the inspired sevoflurane concentration was reduced from 1.0 MAC to 0.34 MAC (i.e., MAC-awake) [15], the estimated time to reach 0.5 MAC [16] was 28 minutes, a period that exceeded ≈95% of surgical closure times for most categories of surgical procedures [8]. The previously studied alarm threshold to prevent recall of 0.5 MAC [16] is suitable based on there being no articles reporting the 90% or 95% effective dose (MAC) to prevent recall (Appendix A). For the aforementioned reasons [8‑13], we therefore expected small to moderate correlations for practitioners’ pairwise MAC goal and MAC achieved. Our secondary objective was to measure the correlation and the effects of covariates on the association.

## Materials and methods

The Vice Chair for Informatics, AAM, emailed all anesthesia practitioners (anesthesiologists, anesthesia residents, and nurse anesthetists) the week before the start of data collection to explain the study [23]. The email provided explanations for how the study would be conducted. It explained that the study’s focus was prolonged extubations, a substantive concern of the department, and a focus of its earlier research [17,18]. The email further noted that the observational design was necessary to obtain relevant data not captured in the electronic health record (Appendices A, B). The practitioners were informed that at the start of surgical closure, the observer, a medical student, would ask them one question about their MAC goal for the end of surgery. (All MAC values in this article are altitude- and age-adjusted; formulas reviewed in Reference [15].) The observer will then repeatedly record the MAC displayed on the anesthesia machine but not information about the patients or practitioners. Because of how cases would be selected, it was unlikely that each would be observed more than once, thereby deliberately making it impossible to compare individuals.

The study inclusion criteria were adult patient (>17 years), inpatient (main) surgical suite, scheduled operating room time at least three hours, tracheal intubation, positioning not prone, regional anesthetic not primary, and no nitrous oxide, isoflurane, desflurane, or amnestic infusion (i.e., propofol, ketamine, or dexmedetomidine). Potential elective cases were reviewed from the operating room schedule the working day before surgery. Potential add-on cases were reviewed on the morning of the day of surgery. All potential cases employed drapes to provide separation of the anesthesia practitioner from the surgical field. To maintain a progressively updated list of the anesthesia practitioners likely to be observed, the scheduled anesthesia practitioner for each suitable case was entered into an Excel 365 web file (Microsoft, Redmond, WA). As noted above, the actual practitioners for the cases were not recorded. The observation was planned for consecutive weeks to achieve balance among weekdays. We expected three cases to be observed daily, progressively decreasing to two or even one cases daily because each practitioner in the study was to be observed for a single case, and many would be excluded as the study progressed. If overall there were approximately 2.5 cases observed per day, then prospective data collection over four weeks (20 days) would obtain approximately 50 cases, matching the necessary sample size. The calculations given in Appendix C, performed using a retrospective cohort of data from the hospital, show that the design and intended sample size were suitable both for assessing variability among practitioners on absolute and relative criteria.

The observer monitored the progress of cases remotely and entered the room before closure began. If the inclusion criteria were satisfied when surgical closure started, the observer asked the anesthesia practitioner, “What is your goal for the age-adjusted MAC when the drapes go down?” The Qualtrics survey tool (Qualtrics XM, Provo, UTAH) was configured for the observer to enter the study data using their phone. The response (i.e., the independent variable) was recorded in 0.1 MAC increments using a slider. The observer also wrote the number on a three-inch-by-three-inch sticky note and put that on the anesthesia machine next to the vaporizer. This was done to increase the salience of the MAC goal and the observer’s role without interruptions. No other questions were asked by the observer. However, if the anesthesia practitioner told the observer the rationale for their MAC goal, that information was saved as a note: both such occurrences were for deep extubation. Neither the use of a Bispectral Index (BIS) monitor (Medtronic, Minneapolis, MN) nor the timing of neuromuscular reversal, with sugammadex at the hospital, was recorded because analyses of a retrospective cohort of data ahead showed that neither influenced the observed MAC and extubation times (Appendices A and B). Changes in MAC were calculated automatically and displayed prominently on the Dräger Medical Perseus A500 anesthesia machines. These MAC values (i.e., dependent variables) were recorded by the observer. The other secondary data recorded were the times of events: start of closure, first dressing on the patient [7], surgical drapes lowered, tracheal extubation, and conversation of any type related to case progress categorized as with or without the estimated minutes remaining. Timestamps of events were recorded by the observer, who tapped corresponding Qualtrics buttons on their phone. Conversations about case progress were counted because the conversations may have led to a more accurate titration of the sevoflurane to achieve the practitioner's MAC goal.

The probability distributions of the MAC goal and MAC achieved were compared with normal distributions using Shapiro-Wilk tests. The ratio of the standard deviations was compared to one using the variance ratio test. Regarding secondary endpoints, the contributions to the associations between the MAC goal and MAC achieved from potential covariates were quantified using the Pearson partial correlation coefficients: the counts of conversations related to case progress were normalized by dividing by the minutes from the start of closure to surgical drapes down, thereby giving rates. (Referring to the raw data [23], this means that if a case has one conversation about the time remaining over the 24 minutes from the start of closure to surgical drapes lowered, the variable used was 0.042, where 0.042 =1/24.) Student’s t‑test with Satterthwaite adjustment for unequal variances was used to compare MAC when surgical drapes were lowered between cases without and with prolonged time to tracheal extubation. For all comparisons, a two-sided P < 0.05 was treated as statistically significant. Stata version 18.0 was used.

## Results

There were N = 70 cases checked in the operating room for potential inclusion to obtain N = 56 meeting inclusion criteria. The practitioners scheduled to be in the operating rooms were 23 anesthesiologists, 19 certified registered nurse anesthetists, and 14 anesthesiology residents, where 56 =23+19+14. The MAC goal and MAC achieved when the surgical drapes were lowered followed normal distributions, with P = 0.90 and P = 0.35, respectively.

**Figure 1:**
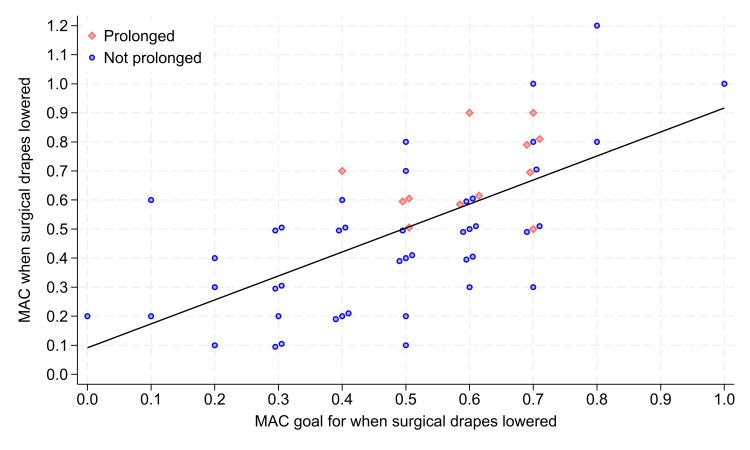
Association among the 56 observations of the anesthesia practitioners’ stated goal minimum alveolar concentration (MAC) for when surgical drapes are lowered versus the MAC achieved at that time The MAC goal was the observer’s only question, asked when surgical closure started. The figure shows the primary endpoint, the variability of the goal among practitioners, along the vertical axis. The anesthesia practitioner told the observer they were doing a deep extubation for two cases, shown at original goal (0.7), achieved (1.0); and original goal (0.8), achieved (1.2), respectively. Excluding the three records with MAC achieved ≥1.0, the estimated ratio of the standard deviation of the goal to the standard deviation of the observed MAC was 0.84, where 0.84 = goal 0.185/ achieved 0.219. The figure also shows the significant association between the two variables: Pearson correlation was 0.65, Spearman correlation was 0.63, and Kendall’s tau = 0.44, all P < 0.0001. The 12 cases (21%) with prolonged extubations (i.e., ≥15 minutes from first dressing on the patient to tracheal extubation) are shown as red triangles, all with MAC achieved ≥0.5.

The standard deviation of the MAC goal among anesthesia practitioners was large, 0.199, with a 95% confidence interval of 0.17 to 0.24 (Figure 1). (The >0.07 threshold standard deviation considered to be “large” is from the Appendix, Section C; P <0.0001 two-sided test compared with 0.07.) There were 5% (3/54) of cases with the MAC goal <0.2 (i.e., low, because less than the estimated MAC for 50% of patients to remember being awake) [19]. There were 25% (14/54) with selected MAC >0.62 (i.e., high, because greater than the estimated MAC for 50% to be awake) [15]. The standard deviation of the MAC goal of 0.199 was not significantly different from the standard deviation of the MAC achieved of 0.253 (Figure 1), P = 0.071. The ratio of the standard deviations of the MAC goal to the MAC achieved was 0.78, where 0.78 = 0.199/0.253. The ratio was sufficiently large that the standard deviation of the MAC goal contributed significantly to the variability in the MAC achieved (Figure 1). If the standard deviation in the anesthesia practitioners' MAC goals had been small, reflecting the limited prior knowledge of the MACs when their patients would awaken (Appendix A) [9‑11], then the ratio of the standard deviations would have been substantively smaller.

Times from dressings on the patients to tracheal extubation were prolonged (i.e., ≥15 minutes) for 21% of cases (12/56; Figure 1). Among the N = 12 cases with prolonged extubation, the mean MAC achieved was 0.60 (0.10) when the drapes went down, significantly larger than among the N = 44 cases without prolonged extubation, 0.48 (0.21), P = 0.0070. The MAC achieved had a moderately large correlation with the MAC goal (Pearson correlation 0.65, P < 0.0001) (Figure 1). Controlling for the MAC goal, there was the absence of significant partial correlations of the MAC achieved at the end of surgery with covariates (Table 1).

**Table 1:** Pearson partial correlation coefficients between MAC achieved and other variables, controlling for MAC goal The mean (standard deviation) of the time from the start of closure to the lowering of the surgical drapes (i.e., the end of surgery) was 42 (26) minutes. The raw data are provided in a supplemental file [23].

Potential covariate	Pearson partial correlation coefficient, P-value
Minutes from start of closure to drapes down	‑0.04, P = 0.78
Rate of operating room conversation(s) of any type related to case progress	-0.01, P = 0.96
Rate of operating room conversation(s), including time remaining	0.19, P = 0.17
Sequence of the N = 56 observations in the study	0.02, P = 0.86

We evaluated what effect the sticky note might have had on the standard deviation of the MAC achieved. The retrospective data had a standard deviation of MAC, for when the surgical dressing was placed, of 0.333, with a 95% confidence interval of 0.316 to 0.350 (Appendix B). The prospective observed standard deviation of MAC, for when the dressing was placed, was comparable, 0.270, with a 95% confidence interval of 0.229 to 0.334; P = 0.058.

## Discussion

The novel feature of our study was asking anesthesia practitioners, at the time that surgical closure began, for their MAC goal at the end of surgery. The results of our study show a considerably larger standard deviation in the sevoflurane MAC goal for the end of surgery among anesthesia practitioners than expected based on the lack of prior knowledge that each practitioner would have for when their patient would awaken (Appendix A) [8‑13]. In other words, although the most common response was as expected (0.5 MAC), most practitioners had a considerably different MAC goal than 0.5, the standard deviation among clinicians being 0.199, much larger than an expected 0.07. Even if clinicians were somehow associating surgical stimulation with patients’ physiological responses at different vaporizer MAC settings, the dose-response for awakening does not match responsiveness during induction and maintenance because of substantial pharmacodynamic hysteresis [10]. The end-tidal MAC needs to be considerably lower for patients to awaken than for patients to lose consciousness at anesthesia induction [10]. A study strength is that the conclusions are especially reliable because we studied sufficiently long-duration cases to have achieved near equilibrium between the inspired and vessel-rich group concentrations of sevoflurane [8].

The study’s secondary results also showed that the MAC goal mattered because practitioners with a larger MAC goal had larger MACs when the drapes were lowered (Figure 1). These larger MACs were associated with a longer time to awaken [9]. Our results were clinically important, as shown by the larger MAC achieved having a significantly greater risk of prolonged extubation (P = 0.0070). In other words, the MAC at the end of surgery mattered clinically and economically.

A strength of our study was that this unadjusted association was supported by the results from the retrospective cohort study (Appendix B), with each 0.1% increase in the MAC when the surgical dressing was placed increasing the adjusted odds of prolonged extubation ≈1.62-fold, P < 0.00001. We showed earlier that providing practitioners with individual feedback on prolonged extubations is not supported because of a lack of statistical reliability [20]. From the current study’s findings (Figure 1), the lack of reliability likely reflected the variability in the ratios of MAC achieved to MAC goal caused by variability in surgical closure time for the procedure [12‑14]. Anesthesia-surgical team communication had no significant effect. Therefore, the future intervention that we recommend to reduce the variability of the MAC goal is education targeting inhalational pharmacodynamics, uptake, and distribution [10,11,15,21]. Essentially, that is as simple as explaining that because there is currently no available data on the MAC-awake for at least 90% of patients (Appendix A), use a MAC goal of 0.5 [16]. In addition, we previously demonstrated in a simulation analysis that increasing the fresh gas flow briefly while decreasing the inspired concentration can help then have the MAC achieved match their MAC goal [8]. We recommend prospectively studying the impact on prolonged extubations of this proposed strategy vs. standard practice to more reliably achieve the MAC goal at the time of surgery end. The long-term effect on the important clinical and economic endpoint of prolonged extubations [1-6] could then be assessed retrospectively, as in Appendix B.

A limitation is that we studied one surgical suite. However, we know from our analysis of the retrospective data that observing each practitioner once was suitable (Appendix B). Also, the mean, median, and mode MAC goals were all 0.50, matching earlier work (Appendix A) and estimates from the retrospective cohort (Appendix C). Results could be different in other training programs, countries, etc., especially because of the impact shown by the MAC goal, the future target focus of teaching and application of the pharmacology of inhalational anesthetics [15,21]. Another limitation was that we studied the MAC goal even if it was high because a deep extubation was planned (Figure 1). However, although doing so increased the standard deviation of the MAC goal, we also analyzed the ratio of the MAC goal to the MAC achieved and obtained the same conclusions (Figure 1).

## Conclusions

Our prospective observational study confirmed that the altitude- and age-adjusted end-tidal MAC at the end of surgery matters because differences of 0.48 versus 0.60 are associated with prolonged extubations. Our novel finding is that these MAC achieved ≥0.60 were partly explained by the anesthesia practitioners’ stated MAC goal. The variability among practitioners in their stated MAC goals was large, relative to evidence-based variability, both on an absolute and relative basis. Therefore, we recommend future interventional studies, to reduce prolonged extubation, examine the benefit of education about inhalational agent pharmacodynamics, uptake, and distribution at the end of surgery.

## Appendices

Appendix A

Literature Search of MAC Alarm Threshold

The previously studied alarm threshold to prevent patient recall was 0.5 MAC [16]. That value was suitable for use because no articles were returned from a PubMed search on June 13, 2024. The search protocol used was: ( ED90[TIAB] OR ED95[TIAB] OR "effective dose 90"[TIAB:~1] OR "effective dose 95"[TIAB:~1] OR "estimated dose 90"[TIAB:~1] OR "estimated dose 95"[TIAB:~1] ) AND ("MAC awake"[TIAB:~1] OR "MAC amnesia"[TIAB:~1] OR "minimum alveolar concentration awake"[TIAB:~1] OR "minimum alveolar concentration amnesia"[TIAB:~1] ).

We relied on MAC-awake. The review article [15] provided no information on temperature adjustment for awakening patients that could be used by anesthesia practitioners as a basis for their evidence-based selection of the MAC goal for the patients in our study. We continued the preceding search for any other related articles. The search protocol used was: ( temperature[TIAB] ) AND ("MAC awake"[TIAB:~1] OR "MAC amnesia"[TIAB:~1] OR "minimum alveolar concentration awake"[TIAB:~1] OR "minimum alveolar concentration amnesia"[TIAB:~1] ). The one article, Eger 2001, provided no formula for or adjustment of MAC-awake for temperature [22].

Appendix B

Analysis of Retrospective Cohort Data to Inform the Prospective Study Design

The prospective observational study was designed using data from a retrospective cohort study of sevoflurane MAC when the circulating nurse documented that the surgical dressing was placed. The following retrospective project, #202308284, was also determined not to meet the regulatory definition of human subjects research because the “activity [was] limited to analysis of de-identified data.”

There were N = 11,703 consecutive cases performed Sunday, April 30, 2023, through Saturday, June 3, 2023, with general anesthesia, tracheal intubation and extubation in the operating room where the anesthetic was performed, and absence of prone positioning. For the subsequent prospective study to include conditions associated with prolonged extubations, we excluded 9353 cases with surgical durations brief enough that less often related to prolonged extubations (operating room entrance to end of surgery less than four hours) [2,5,20], 204 cases with patient age <18 years, 319 cases with mean case desflurane MAC >0.6 or isoflurane MAC >0.6, 173 cases with mean case MAC $\leq$0.6, and 57 cases with concomitant use of nitrous oxide. The resulting sample size of the retrospective cohort was 1607 cases.

Multivariable logistic regression with robust variance estimation was used to evaluate what data needed to be manually recorded prospectively. The incidence of prolonged time to tracheal extubation ($\geq$15 minutes) was 26% (421/1607). Each 0.1% increase in the MAC when the surgical dressing was placed increased the adjusted odds of prolonged extubation 1.62-fold (95% confidence interval 1.025 to 2.563, P < 0.00001). There was no significant association with the use of the BIS monitor (odds ratio $\approx$1.32, P = 0.11), neostigmine (odds ratio $\approx$0.83, P = 0.77), or sugammadex (odds ratio $\approx$0.95, P = 0.64). Therefore, in the prospective study, MAC was recorded, but not BIS or the timing of neuromuscular reversal.

While there was negligible use of neostigmine (1%, 21/1607), the BIS monitor was used often enough (15% of cases, 236/1607) that, although not associated with prolonged extubations, its use could influence the variability in MAC at the end of surgery. Standard deviations of MAC among cases with and without the BIS monitor were compared using robust variance estimated based on the median. The standard deviation with BIS 0.386 versus without BIS 0.322 did not differ significantly, P = 0.27.

Appendix C

Sample Size for the Prospective Study

The sample size needed for the prospective study was designed both using an absolute criterion for the standard deviation of MAC among practitioners and using a comparison compared to the observed standard deviation of MAC at the end of surgery among all the cases.

For the absolute criteria, we expected the standard deviation of the MAC goal to be a small value reflecting the limited prior knowledge of the practitioner for when the patient would awaken [8‑13]. Suppose that 50% of the goals were 0.5 MAC, 25% were 0.4, and 25% were 0.6. Then, the standard deviation of goals would be 0.07. Thus, the statistical power was calculated for comparison of the standard deviation of the MAC goal to 0.07, using Type I error rate alpha = 0.05 and 80% statistical power. Using N = 50 patients would achieve >99% power to detect if the standard deviation of the MAC goal is substantively large (i.e., >0.07).

For the relative criteria, if the standard deviation of the MAC goal was one-third less than the MAC achieved, then the ratio of the goal to realized standard deviation would be 0.666. A two-sided two-group test was planned to compare the ratio of 0.666 to 1.00. Among all 1607 cases, the standard deviation of the MAC was 0.333. A study with N = 50 cases would have 80% statistical power to detect a standard deviation of the MAC goal that is one-third less.

The 0.333 observed standard deviation of MAC when dressing is on the patient has uncertainty. The 95% Bonett confidence interval was 0.316 to 0.350. The necessary sample sizes would then be comparable, 51 or 50 cases, respectively, based on the lower and upper limits. The design also refers to “cases” rather than practitioners. Using a one-way random effects analysis of variance among anesthesia practitioners, the intraclass correlation was 0.13 with a standard error of 0.02. Being small, one estimate per practitioner would reasonably give unbiased estimates for the overall standard deviation.
